# Prediction of fluid responsiveness using respiratory variations in left ventricular stroke area by transoesophageal echocardiographic automated border detection in mechanically ventilated patients

**DOI:** 10.1186/cc5123

**Published:** 2006-12-12

**Authors:** Maxime Cannesson, Juliette Slieker, Olivier Desebbe, Fadi Farhat, Olivier Bastien, Jean-Jacques Lehot

**Affiliations:** 1 Department of Anaesthesiology and Intensive Care, Louis Pradel Hospital, Claude Bernard Lyon 1 university, EA 1896, Hospices Civils de Lyon, Lyon, France; 2 Service de Chirurgie Cardiaque, Hôpital Cardiologique Louis Pradel, 200 avenue du Doyen Lépine, 69500 Bron, France

## Abstract

**Background:**

Left ventricular stroke area by transoesophageal echocardiographic automated border detection has been shown to be strongly correlated to left ventricular stroke volume. Respiratory variations in left ventricular stroke volume or its surrogates are good predictors of fluid responsiveness in mechanically ventilated patients. We hypothesised that respiratory variations in left ventricular stroke area (ΔSA) can predict fluid responsiveness.

**Methods:**

Eighteen mechanically ventilated patients undergoing coronary artery bypass grafting were studied immediately after induction of anaesthesia. Stroke area was measured on a beat-to-beat basis using transoesophageal echocardiographic automated border detection. Haemodynamic and echocardiographic data were measured at baseline and after volume expansion induced by a passive leg raising manoeuvre. Responders to passive leg raising manoeuvre were defined as patients presenting a more than 15% increase in cardiac output.

**Results:**

Cardiac output increased significantly in response to volume expansion induced by passive leg raising (from 2.16 ± 0.79 litres per minute to 2.78 ± 1.08 litres per minute; *p *< 0.01). ΔSA decreased significantly in response to volume expansion (from 17% ± 7% to 8% ± 6%; *p *< 0.01). ΔSA was higher in responders than in non-responders (20% ± 5% versus 10% ± 5%; *p *< 0.01). A cutoff ΔSA value of 16% allowed fluid responsiveness prediction with a sensitivity of 92% and a specificity of 83%. ΔSA at baseline was related to the percentage increase in cardiac output in response to volume expansion (*r *= 0.53, *p *< 0.01).

**Conclusion:**

ΔSA by transoesophageal echocardiographic automated border detection is sensitive to changes in preload, can predict fluid responsiveness, and can quantify the effects of volume expansion on cardiac output. It has potential clinical applications.

## Introduction

Volume expansion is one of the most common manoeuvres to increase cardiac output (CO) in patients with circulatory failure. However, if inappropriate, volume expansion may have deleterious effects such as volume overload, systemic and pulmonary oedema, and increased tissue hypoxia [1]. It is therefore important to obtain reliable information concerning fluid responsiveness in patients presenting with circulatory failure in the operating room or in the intensive care unit.

Static indicators of fluid responsiveness such as central venous pressure (CVP) and pulmonary capillary wedge pressure have been shown to be poor predictors of fluid responsiveness [2-6]. In contrast, indices relying on the cardiopulmonary interactions in mechanically ventilated patients under general anaesthesia have been shown to be good predictors of fluid responsiveness [2-13].

Transoesophageal echocardiography is widely used in the operating room or in the intensive care unit for monitoring left ventricular (LV) systolic function and LV preload [14,15] using LV end-diastolic area index (LVEDAI). However, LVEDAI is a static indicator and has poor predictive value to assess fluid responsiveness [16]. Automated border detection has been shown to be able to display LV area on a beat-to-beat basis, representing the dynamic variant of the left ventricular end-diastolic area (LVEDA) [17,18]. Moreover, through the LV area, the LV volume can be assessed in a non-invasive manner and changes in stroke area (SA) and stroke volume have been shown to be closely related [19-21]. The aim of our study was to evaluate the ability of respiratory variations in LV SA (ΔSA) to detect changes in loading conditions and to predict the effects of volume expansion in mechanically ventilated patients.

## Materials and methods

This study was approved by the institutional review board committee (Comité Consultatif de Protection des Personnes dans la Recherche Biomédicale Lyon B), and all patients gave written informed consent. Twenty patients (aged 37 to 84 years old; 13 men, 7 women) undergoing coronary artery bypass grafting were studied. Exclusion criteria were cardiac arrhythmia, cardiac shunts, LV dysfunction (preoperative LV ejection fraction < 50%), and any contraindication to transoesophageal echocardiography.

Anaesthesia was induced using propofol (1 to 3 mg/kg) and sufentanil (0.5 to 1.0 μg/kg), and orotracheal intubation was facilitated with pancuronium (0.1 to 0.15 mg/kg). After induction of anaesthesia, an 8-cm 5-French tipped catheter (Arrow International, Inc., Reading, PA, USA) was inserted in the left or right radial artery and a triple-lumen 16-cm 8.5-French central venous catheter was inserted in the right internal jugular vein (Arrow International, Inc.). Pressure transducers (Medex Medical Ltd., Rossendale, Lancashire, UK) were placed on the mid-axillary line and fixed to the operation table to ensure their position at an atrial level during the entire protocol. All transducers were zeroed to atmospheric pressure before each step of the protocol. Thereafter, a 5-MHz transoesophageal multiplane transducer (Philips 5.0–6.4 MHz, 21367A; Philips Medical Systems, Andover, MA, USA) was inserted in the patient's oesophagus. Anaesthesia was maintained with continuous infusions of propofol (5 to 8 mg/kg per hour) and sufentanil (0.7 to 1.0 μg/kg per hour) to keep a bispectral index (Aspect 1000; Aspect Medical Systems, Inc., Norwood, MA, USA) between 40 and 50. Patients were ventilated in a volume-controlled mode with a tidal volume of 10 ml/kg at a frequency of 12 to 14 cycles per minute (average maximum inspiratory pressure was 18 ± 5 cm H_2_O). Inspiratory-to-expiratory ratio was set to 1:2. Positive end-expiratory pressure was set between 0 and 4 cm H_2_O according to the attending physician.

### Haemodynamic measurements

The following haemodynamic parameters were monitored continuously (Philips Intellivue MP70 Anaesthesia; Philips Medizin Systeme Böblingen GmbH, Böblingen, Germany): heart rate, systolic arterial pressure, diastolic arterial pressure, mean arterial pressure, and CVP.

### Echocardiography

**Figure 1:**
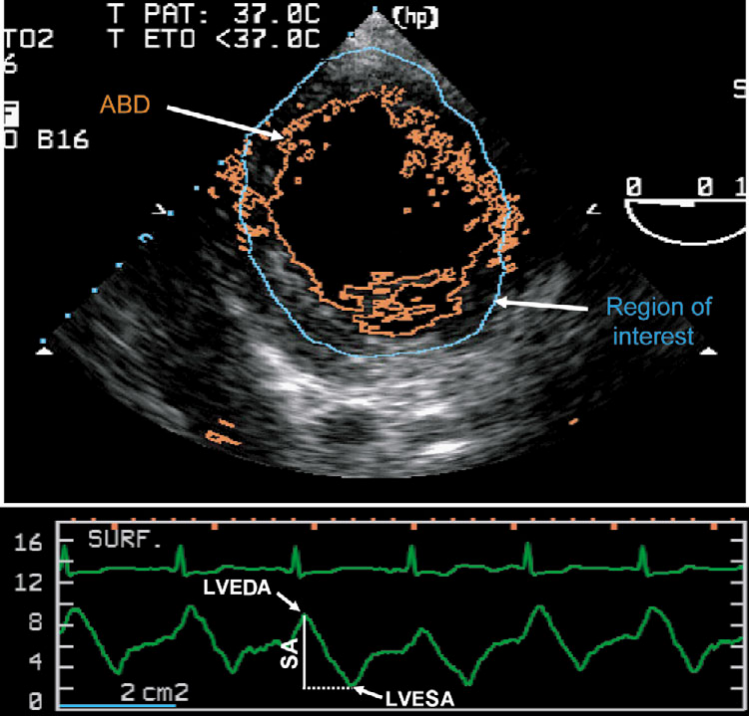
Transoesophageal echocardiographic transgastric, cross-sectional view of the left ventricle at midpapillary muscle level with automated border detection (ABD). Endocardial border of the left ventricle, including the papillary muscles, was circumscribed manually to define the region of interest (blue line). ABD quantifies the cardiac chamber areas instantaneously by detecting the blood-tissue interface (red line), which results in a continuous, beat-to-beat left ventricular area curve (green line). Left ventricular end-diastolic area (LVEDA) was defined as peak of the left ventricular area during diastole. Left ventricular end-systolic area (LVESA) was defined as minimum left ventricular area during systole. Stroke area (SA) was defined as LVEDA – LVESA over the same cardiac cycle.

Echocardiographic images were recorded using a Hewlett-Packard Sonos 2500 (HP M2406A; Hewlett-Packard Company, Palo Alto, CA, USA) with automated border detection capabilities. The transoesophageal multiplane transducer was positioned to obtain a transgastric, cross-sectional view of the LV at midpapillary muscle level with the transducer positioned to obtain the image that had the most-circular overall geometry [22]. The cross-sectional view of the LV was chosen because of a demonstrated relationship between LV cross-sectional area and LV volume [23-25]. Automated border detection quantifies the cardiac chamber areas instantaneously by detecting the tissue-blood interface, which results in a continuous, beat-to-beat ventricular area and has already been described in great detail elsewhere [17-20,26]. Briefly, the endocardial border of the LV, including the papillary muscles, was circumscribed manually to define the region of interest (careful attention was paid to circumscribe the LV all along the respiratory cycle). The threshold for the determination of the blood-tissue border inside this region was set manually by adjusting the gain. The LV area was then displayed on a beat-to-beat basis simultaneously with the patient's electrocardiogram and respiratory curve. It was then recorded and analysed off-line by an investigator blinded to the other results (Figure 1).

### Data analysis

#### Respiratory variations in pulse pressure

Pulse pressure (PP) was defined as the difference between systolic and diastolic pressures. Maximal (PP_max_) and minimal (PP_min_) values of PP were determined over the same respiratory cycle. The respiratory variations in PP, ΔPP, were then calculated as described by Michard and colleagues. [9], ΔPP = [(PP_max _- PP_min_)/([PP_max _+ PP_min_]/2)] × 100%, and averaged over three consecutive respiratory cycles.

#### Respiratory variations in stroke area

**Figure 2:**
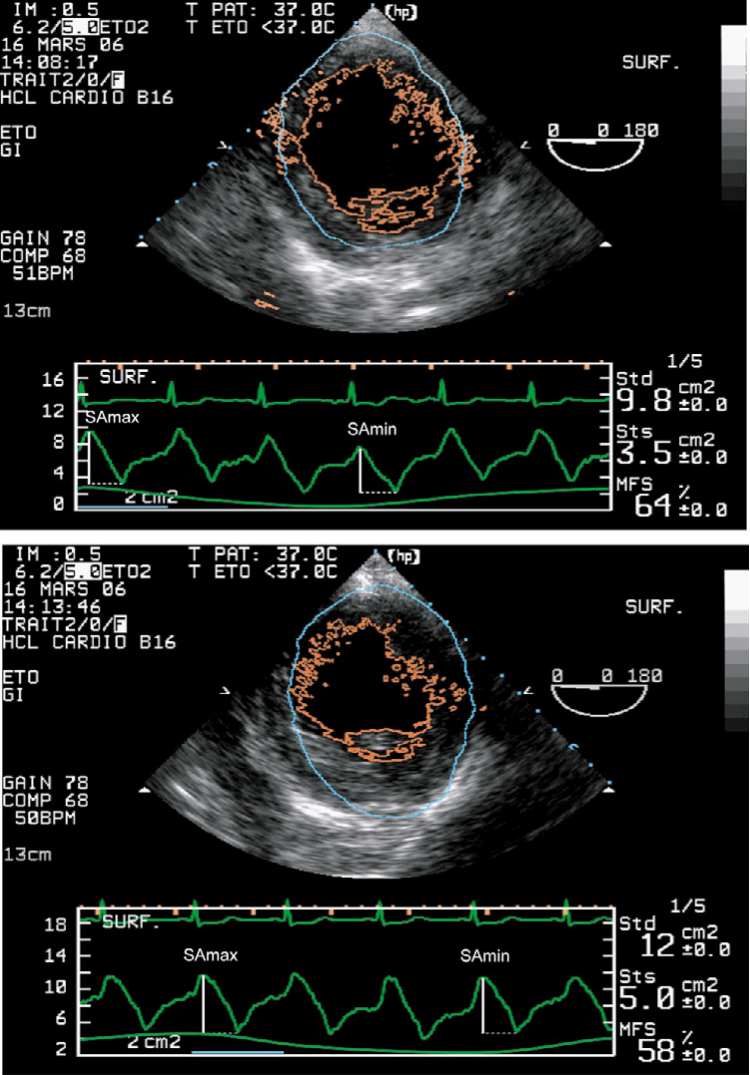
Transoesophageal echocardiographic transgastric, cross-sectional views of the left ventricle at midpapillary muscle level with automated border detection at baseline (top panel) and after volume expansion induced by passive leg raising manoeuvre (bottom panel). Left ventricular area curve was displayed with electrocardiogram and respiratory curve. Stroke area (SA) was defined as the difference between the end-diastolic area (LVEDA) and the end-systolic area. Maximal (SA_max_) and minimal (SA_min_) values of pulse pressure were determined over the same respiratory cycle. Respiratory variations in left ventricular SA (ΔSA) were then calculated using the following formula: ΔSA = [(SA_max _- SA_min_)/([SA_max _+ SA_min_]/2)] × 100%. Passive leg raising manoeuvre induced a decrease in ΔSA and an increase in LVEDA. Gain was held constant throughout the protocol.

SA was defined as the difference between the end-diastolic area (LVEDA) and the end-systolic area (Figure 1) [20]. Maximal (SA_max_) and minimal (SA_min_) values of PP were determined over the same respiratory cycle. ΔSA was then calculated using the same formula described previously to calculate ΔPP: [(SA_max _- SA_min_)/([SA_max _+ SA_min_]/2)] × 100% (Figure 2). ΔSA and ΔPP were calculated over the same respiratory cycles.

#### Left ventricular end-diastolic area

LVEDA was defined as peak of the LV area during diastole. LVEDAI was defined as LVEDA/surface body area. For each measurement, an average of three consecutive cardiac beats throughout the respiratory cycle were evaluated.

#### Cardiac output

CO was used to monitor an increase in stroke volume in response to volume expansion. CO was calculated using the velocity time integral (VTI) obtained by transoesophageal echocardiography from the transgastric long-axis view [27]. VTI was measured by a pulsed-wave Doppler beam at the level of the aortic valve. The mean of three measurements was used. Aortic valve area was measured at baseline and was considered constant throughout the protocol as aortic valve area = π × (aortic diameter/2)^2^. [4,28]. The stroke volume was calculated as aortic valve area × VTI. CO was calculated as stroke volume × heart rate.

### Protocol

All patients were studied after induction of anaesthesia but before surgery. Haemodynamic and echocardiographic data were recorded during two consecutive steps. (a) The patient was studied in the semirecumbent position (45°) after a two minute period of haemodynamic stability. First, pulsed Doppler aortic flow was recorded from the transgastric long-axis view. Then, automated border detection data were recorded from a transgastric, cross-sectional view of the LV at midpapillary muscle level. (b) Using the automatic operation table, the patient's legs were raised to a 45° angle with the patient's trunk in a supine position. In this position, echocardiographic and haemodynamic data were recorded within two minutes. Automated border detection data were recorded before pulsed Doppler aortic flow. This sequence was chosen in order to keep the automated border detection settings stable between the two measurements because of a known dependency of automated border detection on echocardiographic gain settings. This protocol was chosen because of its demonstrated ability to mimic fluid challenge [29-32]. Mechanical ventilation and anaesthetic drug concentrations were held constant throughout the study protocol.

### Statistical analysis

All data are presented as mean ± standard deviation. Changes in haemodynamic parameters induced by changes in loading conditions within the same group were assessed using a non-parametric Wilcoxon test. Spearman rank method was used to test linear correlation. Patients were divided into two groups according to the percentage increase in CO after the passive leg raising manoeuvre: responders were defined as patients presenting an increase in CO of more than or equal to 15% [9] and non-responders as patients presenting an increase in CO of less than 15%. The comparison of haemodynamic parameters before passive leg raising in responder and non-responder patients was assessed using a non-parametric Mann-Whitney *U *test. Receiver operating characteristic (ROC) curves were generated for CO, CVP, LVEDA, ΔPP, and ΔSA, varying the discriminating threshold of each parameters, and areas under the ROC curves were calculated and compared [33] (MedCalc 8.0.2.0; MedCalc Software, Mariakerke, Belgium). Intra- and interobserver variabilities for the calculation of ΔSA were assessed using Bland-Altman analysis and are expressed as mean percentage error [34]. This analysis comprised visualisation and re-installation of the automated border detection in nine patients at baseline by two different operators. A *p *value less than 0.05 was considered statistically significant. All statistic analysis was performed using SPSS 13.0 for Windows (SPSS Inc., Chicago, IL, USA).

## Results

Two patients (10%) were excluded because of poor echocardiographic images.

### Effects of changes in loading conditions

As expected, passive leg raising induced a significant increase in CO, from 2.16 ± 0.79 litres per minute to 2.78 ± 1.08 litres per minute (*p *< 0.01). All haemodynamic parameters changed significantly in response to the passive leg raising manoeuvre (Table 1). ΔSA and ΔPP decreased significantly in response to passive leg raising (from 17.1% ± 6.8% to 8.1% ± 5.8% and from 9.9% ± 5.5% to 7.9% ± 3.2%, respectively; *p *< 0.05 for both) (Figure 2). Likewise, LVEDAI increased from 9.2 ± 4.5 cm^2^/m^2 ^to 10.8 ± 6.3 cm^2^/m^2 ^(*p *< 0.05) and CVP increased from 3 ± 4 mm Hg to 18 ± 4 mm Hg (*p *< 0.01) (Figure 2).

### ΔSA to predict fluid responsiveness

**Figure 3:**
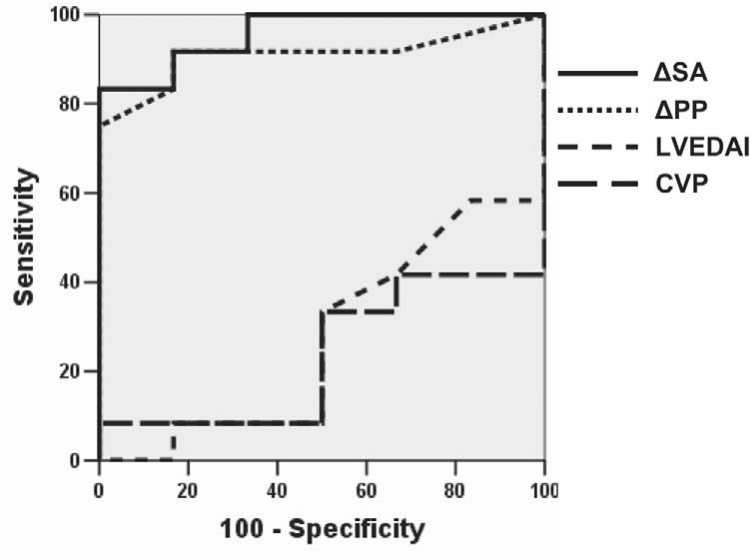
Receiver operating characteristic curves comparing the ability of respiratory variations in left ventricular stroke area (ΔSA), respiratory variations in pulse pressure (ΔPP), left ventricular end-diastolic area index (LVEDAI), and central venous pressure (CVP) at baseline to predict response to volume expansion induced by passive leg raising manoeuvre.

**Figure 4:**
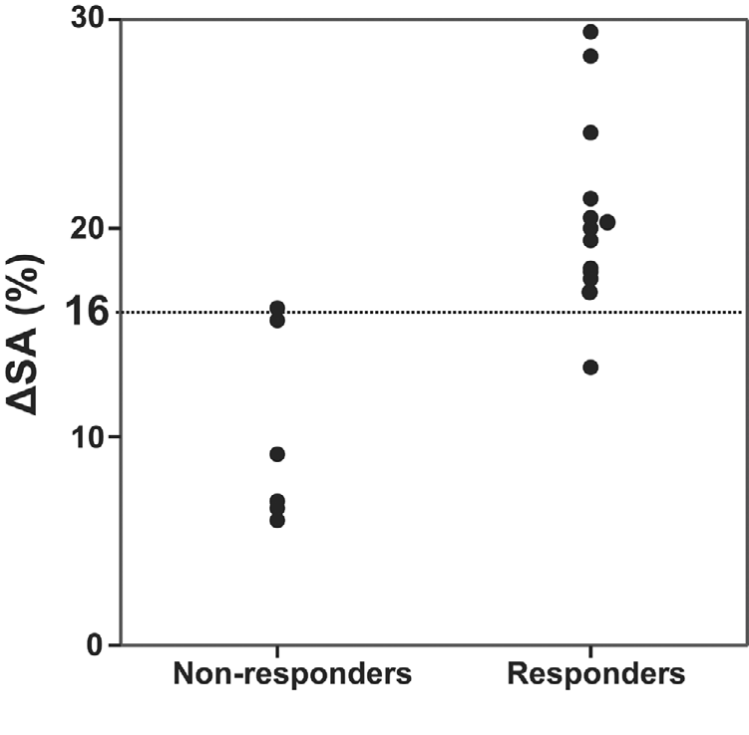
Respiratory variations in stroke area (ΔSA) values at baseline in responders and non-responders to volume expansion induced by passive leg raising manoeuvre. A ΔSA threshold value of 16% allowed discrimination between responders and non-responders with a 93% sensitivity and an 82% specificity.

Twelve patients were responders and six patients were non-responders. Their haemodynamic data are shown in Table 2. We observed a significant relationship (*r *= 0.62, *p *< 0.05) and an acceptable agreement between ΔSA and ΔPP (3% ± 5%) at baseline. ΔSA and ΔPP at baseline were significantly higher in responders than in non-responders (20.5% ± 4.8% versus 10.0% ± 4.6% and 17% ± 5% versus 8% ± 4%; *p *< 0.01 for both), whereas neither difference in CVP (6 ± 3 mm Hg in responders versus 9 ± 4 mm Hg in non-responders; *p *= 0.13) nor difference in LVEDAI (7.9 ± 4.1 cm^2^/m^2 ^versus 11.9 ± 4.5 cm^2^/m^2^; *p *= 0.06) and CO (2.17 ± 0.94 litres per minute in responders versus 2.14 ± 0.43 litres per minute in non-responders; *p *= 0.94) reached statistical significance between these two groups. The areas under the ROC curves (± standard error) were 0.910 ± 0.073 for ΔPP, 0.958 ± 0.043 for ΔSA, 0.271 ± 0.125 for CVP, 0.236 ± 0.114 for LVEDAI, and 0.431 ± 0.134 for CO (Figure 3). The area for ΔSA was significantly higher than the area for CVP, LVEDAI, and CO (*p *< 0.05). Difference in area under the curve between ΔSA and ΔPP did not reach significance (*p *= 0.83). The threshold ΔPP value of 12% allowed discrimination between responders and non-responders with a sensitivity of 92% and a specificity of 83%. The threshold ΔSA value of 16% allowed discrimination between responders and non-responders with a sensitivity of 92% and a specificity of 83% (Figure 4).

### ΔSA to quantify response to volume expansion

**Figure 5:**
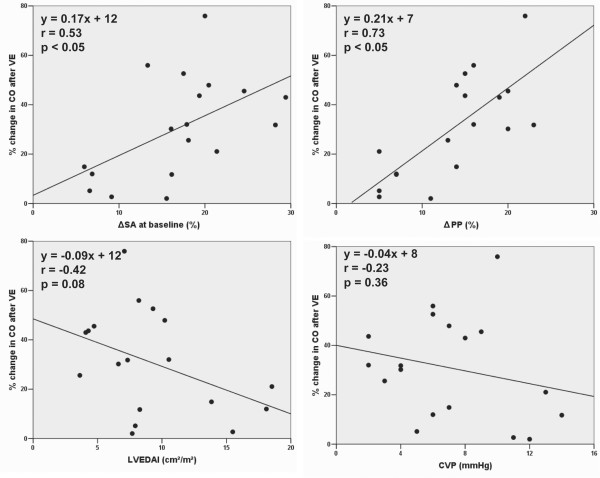
Relationship between respiratory variations in left ventricular stroke area (ΔSA) (top left panel), respiratory variations in pulse pressure (ΔPP) (top right panel), left ventricular end-diastolic area index (LVEDAI) (bottom left panel), and central venous pressure (CVP) (bottom right panel) at baseline and percentage increase in cardiac output (CO) after volume expansion (VE) induced by passive leg raising manoeuvre.

ΔSA before volume expansion was significantly related to changes in CO in response to volume expansion (*r *= 0.53, *p *< 0.05). ΔPP before volume expansion also showed a significant correlation to changes in CO (*r *= 0.73, *p *< 0.01), confirming previous results. In contrast, static indicators such as LVEDAI and CVP before volume expansion were not related to changes in CO in response to volume expansion (*r *= -0.42, *p *= 0.08 and *r *= -0.23, *p *= 0.36, respectively) (Figure 5).

### Reproducibility analysis

Intraobserver variability for ΔSA assessment was 8% ± 12%. Interobserver variability for ΔSA assessment was 10% ± 12%.

## Discussion

This is the first study to show that ΔSA can be assessed using automated border detection. ΔSA is sensitive to changes in LV loading conditions, can predict and quantify fluid responsiveness, and is reproducible.

Fluid responsiveness assessment has been widely studied in mechanically ventilated patients during the past 10 years [2-13,28,31,35,36]. Positive pressure ventilation induces a decrease in right ventricular preload during inspiration followed by a decrease in right ventricular stroke volume (as described by the Frank-Starling relationship). These phenomena are transmitted to the LV (pulmonary transit time) and induce a decrease in LV preload followed by a decrease in LV stroke volume during expiration [2,37]. These respiratory variations in LV stroke volume or its surrogates are greater when the LV operates on the steep portion of the Frank-Starling curve rather than on the plateau. These phenomena explain how the respiratory variations in LV stroke volume or its surrogates (PP, pulsed Doppler aortic flow) can be predictive of response to volume expansion [2]. Indices derived from these respiratory variations are qualified as dynamic predictors of fluid responsiveness in opposition to static predictors such as CVP, pulmonary capillary wedge pressure, or LVEDAI [2,37]. Moreover, it is now well established that dynamic indicators have better predictive value for fluid responsiveness assessment than do static indicators alone [2,37].

Automated border detection allows accurate and reproducible on-line measurements of cross-sectional LV area. It analyses received unprocessed radio frequency data to define the interface between blood and myocardial tissue. Then, the software calculates the blood cavity area within a specified region of interest (LV) and displays the area as a calibrated waveform in real time. Several previous investigations have shown a strong relationship between cross-sectional LV area and LV volume [18,23]. Moreover, this relationship was demonstrated during a wide range of haemodynamic alterations such as occlusion and release of inferior vena cava, pulmonary artery, and aorta. By displaying LV area continuously, automated border detection allows beat-to-beat determination of LV SA (defined as LV end-diastolic area – LV end-systolic area). LV SA has been shown to be closely related to LV stroke volume [20,21,26,38], and this relationship has been demonstrated in various ventricular loading conditions [20,21] and in patients with wall motion abnormalities [21]. Coupled to LV pressure, LV cavity area has been proposed to construct pressure-area loops in real time in order to estimate LV contractility from end-systolic relationships of cavity area (as a surrogate for LV volume) and central arterial pressure (as a surrogate for LV pressure) with promising results [39]. Gorcsan and colleagues [20,21] have shown that changes in LV stroke volume during vena cava occlusion were strongly related to changes in LV SA in patients undergoing coronary artery bypass surgery and in dogs. It must be emphasised that these changes were studied in a beat-to-beat analysis. Thus, changes in preload can be assessed using automated border detection LV SA. Our results are consistent with this previously published data given that changes in preload induced by positive pressure ventilation were quantifiable using respiratory changes in LV SA (ΔSA). Furthermore, these variations were reduced after a volume expansion induced by a passive leg raising manoeuvre and were higher in responders to volume expansion than in non-responders. Of note, in 1978, Brenner and colleagues [40] were the first to describe respiratory changes in LV dimensions using echocardiography. In a study focusing on spontaneously breathing patients with normal ventricular function, they showed respiration-induced changes in LV end-diastolic and end-systolic diameters measured from a parasternal mid-short-axis view using M-mode. It is interesting to note that in this study the authors showed an inspiratory decrease in LV stroke volume. In mechanically ventilated patients, we observed an inspiratory increase in LV SA consistent with the cardiopulmonary interactions in patients under positive pressure ventilation [2]. However, the relationship between mechanical ventilation, arterial pressure, and LV area and volume is still complex and some studies found an inconstant association between respiration-induced changes in systolic arterial pressure and changes in LV area [41].

Recently published studies have shown that the passive leg raising manoeuvre was able to mimic volume expansion and to predict fluid responsiveness in mechanically ventilated patients [31,32]. These two studies show that patients who significantly increase CO after a passive leg raising manoeuvre are more likely to be responders to volume expansion. The major interest of this manoeuvre is that it can be performed in patients with arrhythmia, even if the patient is triggering on the ventilator in the intensive care unit. However, the passive leg raising manoeuvre may not be easy to perform in the operating room in patients undergoing surgical procedures with surgeons needing exposure and access to the operating field.

Transoesophageal echocardiography is widely used in the intensive care unit and in the operating room. It is now a well-established tool for intensivists and anaesthesiologists. It allows analysis of left and right ventricular functions and provides invaluable information for the management of patients with circulatory failure [15,27]. In the intensive care unit, echocardiography is a useful tool to assess fluid responsiveness (respiratory variations in pulsed Doppler aortic blood velocity, inferior vena cava diameter, and superior vena cava collapsibility) [42]. In this setting, automated border detection could be used as a new tool to discriminate between responders to volume expansion and non-responders. In the operating room, transoesophageal echocardiography has been proposed for LV systolic function and LV preload monitoring [16,35,43,44]. Monitoring preload is different from assessing fluid responsiveness, and LVEDAI has been shown to poorly predict response to volume expansion. Using automated border detection and ΔSA in this setting may be helpful to monitor both systolic function and fluid responsiveness from a transgastric, cross-sectional view of the LV at midpapillary muscle level.

### Study limitations

The patients enrolled in this study underwent coronary artery bypass grafting and may have wall motion abnormalities. However, a study conducted in patients undergoing coronary artery bypass grafting demonstrated that even in this group of patients a linear correlation exists between changes in SA and stroke volume [21]. Thus, we are confident that wall motion abnormalities had no influence on ΔSA. We performed passive leg raising with trunk lowering from 45° to 0° to mimic volume expansion as described by Monnet and colleagues[31]. This manoeuvre has been shown to be a simple method of transiently increasing venous return [29,30] and has recently been shown to be able to predict fluid responsiveness in mechanically ventilated patients [31]. Moreover, echocardiographic data were obtained within two minutes after passive leg raising because it is known that the fluid challenge induced by passive leg raising does not persist if legs are maintained elevated. This is in accordance with previously published studies [31,32]. We chose to use a standardised tidal volume of 10 ml/kg because it has been demonstrated that tidal volume influences dynamic parameters [45,46]. Most of the studies focusing on dynamic parameters in the operating room chose to use tidal volumes between 8 and 10 ml/kg. Thus, we believe that our choice is in accordance with these studies.

A limitation of this study is a possible artifact caused by a respiratory-related motion of the heart relative to a fixed echocardiographic probe. Brenner and colleagues [40] described this hypothesis without being able to reject it. In our study, we cannot exclude such an artifact, which would result in a different LV short axis during the respiratory cycle and influence the respiratory changes in end-diastolic and end-systolic area, especially during the passive leg raising manoeuvre. This limitation may be observed for most of the previously described echocardiographic predictors of fluid responsiveness because respiration may move either the ultrasound beam or the studied structure (inferior [28] or superior [36] vena cava diameter, LV outflow tract for aortic pulsed Doppler flow [4]). However, the respiratory changes in LV SA are consistent with previously published respiratory changes in LV stroke volume or its surrogates in patients under mechanical ventilation and we can postulate that ΔSA accurately reflects respiratory changes in LV stroke volume. In our study, passive leg raising may have induced displacement of the transoesophageal probe. On the other hand, from a prospective point of view, we used ΔSA before passive leg raising to predict fluid responsiveness. Consequently, this index was not influenced by change in body position. Thus, the predictive value of ΔSA is not impacted by this manoeuvre. Moreover, previously published studies focusing on oesophageal Doppler and passive leg raising did not mention this technical problem [31,32]. An additional limitation is that automated border detection is dependent on the gain setting. However, we held the gain constant throughout the protocol. Whether ΔSA can be assessed using transthoracic echocardiography has to be demonstrated. The results were obtained from 18 patients and the study is underpowered to permit a definitive conclusion regarding the threshold value of 16%. Further studies in other settings will be required to validate this value. Finally, ΔSA cannot be used in spontaneously breathing patients or in patients with cardiac arrhythmia.

## Conclusion

ΔSA derived from transoesophageal echocardiographic automated border detection appears to be a non-invasive and reproducible index of changes in loading conditions, fluid responsiveness, and quantification of the effects of volume expansion on CO in mechanically ventilated patients. ΔSA has potential clinical applications.

## Key messages

• ΔSA can be assessed using transoesophageal echocardiographic automated border detection in mechanically ventilated patients.

• ΔSA is sensitive to changes in LV preload.

• A ΔSA cutoff value of 16% allows discrimination between responders to volume expansion and non-responders.

• The higher the ΔSA before volume expansion, the higher the increase in CO induced by volume expansion.

• ΔSA is a simple tool for fluid responsiveness assessment using transoesophageal echocardiographic LV short-axis view.

## Abbreviations

CO = cardiac output; CVP = central venous pressure; LV = left ventricle or left ventricular; LVEDA = left ventricular end-diastolic area; LVEDAI = left ventricular end-diastolic area index; PP = pulse pressure; ΔPP = respiratory variations in pulse pressure; ROC = receiver operating characteristic; SA = stroke area; ΔSA = respiratory variations in left ventricular stroke area; VTI = velocity time integral.

## Competing interests

The authors declare that they have no competing interests.

## Authors' contributions

MC conceived of and designed the study, performed analysis and interpretation of data, edited the manuscript, and gave final approval of the manuscript. JS performed analysis and interpretation of data, drafted the manuscript, and gave final approval of the manuscript. OD and FF performed analysis and interpretation of data and gave final approval of the manuscript. OB and J-JL revised the manuscript critically for important intellectual content, edited the manuscript, and gave final approval of the manuscript.

## Figures and Tables

**Table 1:** Haemodynamic data at baseline and after volume expansion induced by passive leg raising manoeuvre

	Baseline	Passive leg raising	*p*
ΔSA (percentage)	17 ± 7	8 ± 6^a^	< 0.001
ΔPP (percentage)	9.9 ± 5.5	7.9 ± 3.2^a^	0.003
Central venous pressure (mm Hg)	3 ± 4	18 ± 4^a^	0.005
LVEDAI (cm^2^/m^2^)	9.2 ± 4.5	10.8 ± 6.3^a^	0.02
Heart rate (beats per minute)	64 ± 18	61 ± 16	0.092
Mean arterial pressure (mm Hg)	58 ± 10	74 ± 11^a^	0.002
Velocity time integral (cm)	12.0 ± 4.1	15.9 ± 4.7^a^	< 0.001
Cardiac output (litres per minute)	2.16 ± 0.79	2.78 ± 1.08^a^	0.008

Data are presented as mean ± standard deviation. LVEDAI, left ventricular end-diastolic area index; ΔPP, respiratory variations in pulse pressure; ΔSA, respiratory variations in left ventricular stroke area. ^a ^*p *< 0.05 compared to baseline.

**Table 2:** Echocardiographic and haemodynamic data in responders and non-responders to volume expansion induced by passive leg raising manoeuvre

	Responders (*n *= 12)	Non-responders (*n *= 6)	*p*
ΔSA (percentage)	20 ± 5	10 ± 5	0.001
ΔPP (percentage)	16.5 ± 4.8	8.2 ± 3.6	0.001
Central venous pressure (mm Hg)	6.2 ± 3.4	9.2 ± 3.6	0.13
LVEDAI (cm^2^/m^2^)	7.9 ± 4.1	11.9 ± 4.5	0.10
Cardiac output (litres per minute)	2.17 ± 0.95	2.14 ± 0.43	0.93
Heart rate (beats per minute)	65 ± 17	63 ± 18	0.91

Data are presented as mean ± standard deviation. LVEDAI, left ventricular end-diastolic area index; ΔPP, respiratory variations in pulse pressure; ΔSA, respiratory variations in left ventricular stroke area.
